# High-Resolution Profiling of the Functional Heterogeneity of Technical Lignins

**DOI:** 10.1021/acs.biomac.1c01630

**Published:** 2022-02-25

**Authors:** Oliver Musl, Samira Galler, Gerhild Wurzer, Markus Bacher, Irina Sulaeva, Ivan Sumerskii, Arnulf Kai Mahler, Thomas Rosenau, Antje Potthast

**Affiliations:** † Department of Chemistry, Institute of Chemistry of Renewable Resources, University of Natural Resources and Life Sciences, Konrad-Lorenz-Strasse 24, A-3430 Tulln, Austria; ‡ Core Facility “Analysis of Lignocellulose” ALICE, University of Natural Resources and Life Sciences, Konrad-Lorenz-Strasse 24, A-3430 Tulln, Austria; § Sappi Europe, Sappi Papier Holding GmbH, Bruckner Straße 21, A-8101 Gratkorn, Austria

## Abstract

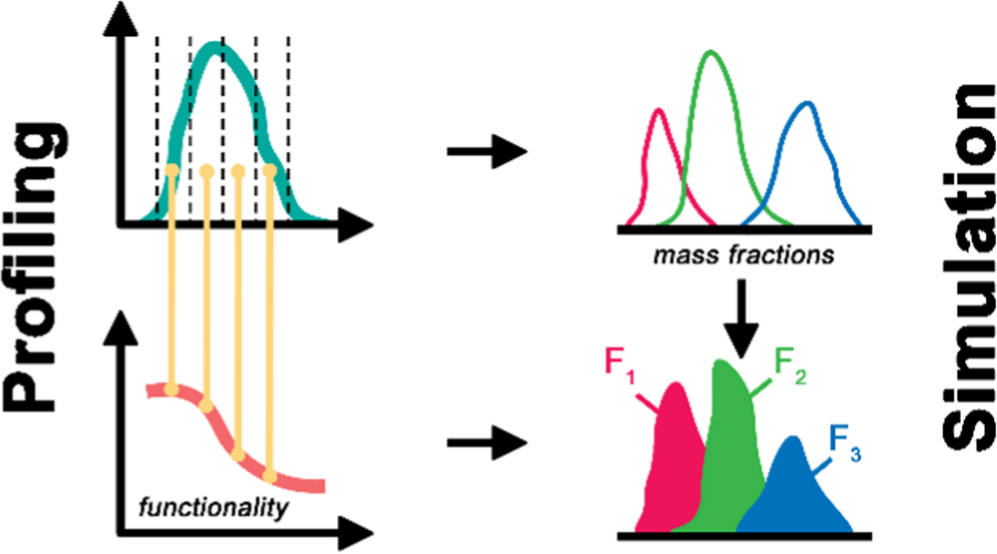

In technical lignins, functionality is strongly related to molar mass. Hence, any technical lignin exhibits concurrent functionality-type distribution (FTD) along its molar mass distribution (MMD). This study combined preparative size-exclusion chromatography with offline characterizations to acquire highly resolved profiles of the functional heterogeneity of technical lignins, which represent crucial information for their material use. The shape of these profiles showed considerable dissimilarity between different technical lignins and followed sigmoid trends. Determining the dispersity in functionality (*Đ*_F_) of lignins via their FTD revealed a rather homogeneous distribution of their functionalities (*Đ*_F_ of 1.00–1.21). The high resolution of the acquired profiles of functional heterogeneity facilitated the development of a robust calculation method for the estimation of functional group contents of lignin fractions based simply on their MMD, an invaluable tool to simulate the effects of intended purification processes. Moreover, a more thorough evaluation of separations based on functionality becomes accessible.

## Introduction

Technical lignins are important co-products of industrial pulping processes. Traditionally, only kraft and sulfite processes are relevant in the pulp and paper industry, with an estimated annual production of 55 million and 1 million tons of technical lignins, respectively.^1^ Although the potential availability of huge quantities draws a great deal of attention, kraft lignins are not yet commercialized for material use on a regular basis, while lignosulfonates are already marketed for a broad set of applications.^1−3^ In both cases, well-developed analytical procedures are desperately needed for adequate elucidation of their complex structural composition, which, due to their heterogeneity and intricacy, represents a major impediment in industrial production. Technical lignins attribute their complex composition, in part, to the natural variability of native lignins, showing differences even at the species level.^4^ Especially, the structural differences between hard and softwood lignins—the main raw materials for industrial pulping—are substantial.^4,5^ However, extreme changes in the native lignin structure are certain to occur in the harsh environment of industrial pulping. In the kraft process, the original lignin structure is vigorously transformed by fragmentation and condensation processes,^6^ whereas the sulfite process is mainly characterized by extensive sulfonation of the lignin backbone.^7^ In either case, the distinctive composition of technical lignin is governed by the impact of the pulping process, including process stages, process conditions, cooking chemicals, catalysts, process intensity, and so on.

For these reasons, technical lignins constitute complicated polymer mixtures showing a distribution in molar mass (molar mass distribution [MMD]) as well as in functionality (functionality-type distribution [FTD]). In this context, functional heterogeneity describes the correlation between molar mass and functionality.^8,9^ Currently, the description of lignin composition focuses mainly on parameters, such as the weight-average molar mass (*M*_w_) or the average content of specific functional groups, which reflect only a part of the underlying dispersity of these structural characteristics. A primary tool for determining the MMD is size-exclusion chromatography (SEC), in which, ideally, the separation of macromolecules is based solely on the hydrodynamic radii of the solutes. Lignins are difficult solutes in SEC because their solubility is limited in common solvents and interactions with the stationary phase have to be suppressed by the addition of salts.^10,11^ In addition, implementing reliable detection systems can be troublesome.^12^ For SEC of kraft lignins, organic solvents or alkaline solutions serve as the mobile phase, while for lignosulfonates, aqueous buffer systems are more common.^10,11^ Although the solubility of (kraft) lignins in ammonium hydroxide solutions is well known, they are not regularly used as a mobile phase in lignin SEC. In contrast, studies on the FTD of lignins typically rely on solvent fractionation or ultrafiltration of lignins followed by functional group characterization.^6,13−18^ Both methods suffer from limited flexibility—a limited number of solvents or membrane cutoffs—resulting in higher dispersity in molar mass within the fractions compared to (preparative) SEC.^19^ In any case, the low molar mass fraction was found to be associated with higher functionality in general, exhibiting a higher content of aromatic hydroxy and carboxylic acid groups (or sulfonic acid groups, in the case of lignosulfonates) than the high molar mass fraction, whereas the opposite was true for aliphatic hydroxy groups.^14,15,20−25^ This may not apply to every technical lignin.^17,26,27^ However, a highly resolved course of functional group contents along the molar mass range has not been achieved to date for a broader selection of technical lignins. The aim of this study was to establish a versatile SEC system for the preparative fractionation of kraft lignins and lignosulfonates to gain deeper insight into their functional heterogeneity (i.e., the molar mass-dependent profile in functional group content). In addition, we attempted to provide a valuable calculation tool to simulate the impact of purification steps on the functional group content of the resulting fractions and a calculation tool that offers an improved method for evaluating the selectivity of separation processes in terms of functionality composition.

## Experimental Section

### Raw Material

Five technical lignins were studied, including three lignosulfonates and two kraft lignins. Lignosulfonates were extracted from industrial sulfite spent liquors originating from different processes—HWLS, a hardwood (beech) Mg lignosulfonate; SWLS, a (mainly) softwood Mg lignosulfonate; and HWNSSC, a hardwood (eucalyptus) Na lignosulfonate from a neutral sulfite semi-chemical (NSSC) pulping process. Both Mg sulfite spent liquors were purified according to Sumerskii et al.^28^ using Amberlite XAD-7 (20–60 mesh), a macroporous polyacrylate resin, and Dowex 50WX8, a strongly acidic cation exchange resin. Both resins were obtained from Sigma-Aldrich and pretreated as described by Sumerskii et al.^28^ The purification process removes carbohydrate-derived and inorganic components of the sulfite spent liquor. NSSC spent liquor was purified by ultrafiltration, which was carried out using a 200-mL ultrafiltration cell (Amicon, Model 8200, Merck Millipore, Billerica) and an Ultracel regenerated cellulose (RC) membrane from Merck Millipore (Billerica) with a cutoff of 1 kDa (230 μm thickness; 63.5 mm diameter). Filtration was performed in deionized water under nitrogen (2.5–3.0 bar) at room temperature.

Kraft lignin was extracted from industrial black liquor by acid precipitation—HWKL, a (mainly) hardwood kraft lignin. Acid precipitation was carried out using 1 M HCl, acidified water was used for washing, and centrifugation was applied to enhance sedimentation. For SWKL, commercially available softwood (pine) kraft lignin Indulin AT (MeadWestvaco) was used without purification.

### Mobile Phases

For lignosulfonates, a 50 mM ammonium chloride (NH_4_Cl) solution was prepared by dissolving 2.375 g of NH_4_Cl in 1 L of water (HPLC grade). A 2 M ammonium hydroxide (NH_4_OH) solution was used to adjust the pH to 9. Sodium azide (NaN_3_), 0.1 g/L, was added against microbial growth. For kraft lignin, a 2 M NH_4_OH solution was prepared by diluting 130 mL of 28–30% NH_4_OH in 1 L of water, resulting in a pH of 12. All eluents were filtered through a 0.2 μm membrane (VacuCap 60 filter unit, Pall Corporation, Port Washington, NY). Water (HPLC grade), NH_4_Cl (>99.5%), NaN_3_ (>99.5%), and NH_4_OH (28–30%, HPLC grade) were purchased from Sigma-Aldrich-Fluka-Merck (Schnelldorf, Germany) and used without further purification.

### Preparative SEC Setup

The preparative HPLC system consisted of an 1800 binary low-pressure gradient pump (250 mL min^–1^ pump head); a preparative 5.9 mL mixing chamber; an ASM 2.1 L sample-loading pump (50 mL min^–1^ pump head) for sample application (all Knauer, Berlin, Germany); a three-way, six-port valve to switch between pumps; and a 1:20 (v/v) fixed-ratio splitter (ERC, Riemerling, Germany). The system was equipped with a preparative SEC column (MCX, molar mass range 1–1 000 kDa, 20 × 300 mm) from Polymer Standard Service GmbH (Mainz, Germany). An Azura UVD 2.1 S detector was used for UV detection at 280 nm (Knauer, Berlin, Germany). The fractions were collected on an ISCO FOXY R1 with a 36-position funnel rack (Teledyne, Lincoln, NE). Clarity Chrom software V8.1.0 (Knauer, Berlin, Germany) was used to control the chromatographic system and data acquisition.

For sample preparation, 5 g of purified lignin was dissolved in 250 mL of the respective mobile phase, shaken overnight, and finally filtered through a 0.45 μm PTFE syringe filter. The HWNSSC was filtered through 0.45 μm cellulose acetate membrane filters (Ø 47 mm; Sartorius Stedim Biotech GmbH, Göttingen, Germany) due to repulsion in the PTFE filters. The effective sample concentration was determined by drying 2 mL of sample solution overnight at 105 °C.

The sample solution (8 mL, 190–270 mg lignin) was loaded onto the column at 10 mL min^–1^ with the loading pump. The flow rate was set to 6 mL min^–1^. The fractionation took place at room temperature. In total, 18–20 fractions were collected per lignin. The sampling intervals were adapted according to the molar mass distribution of the respective lignin. After pooling the fractions, sample purification was carried out by evaporation and lyophilization. In the case of lignosulfonates, excess 1 M sodium hydroxide (NaOH) was added to eliminate ammonia (NH_3_) during evaporation. Then, ion exchange using Dowex 50WX8 was performed to eliminate sodium before lyophilization.

### Nuclear Magnetic Resonance (NMR) Spectroscopy

All NMR spectra were recorded on a Bruker Avance II 400 or a Bruker Avance III HD 400 (resonance frequencies 400.13 and 100.63 MHz for ^1^H and ^13^C) equipped with a 5 mm broadband observe probe head (BBFO) or a liquid N_2_-cooled cryoprobe head (Prodigy) with z-gradients at room temperature with standard Bruker pulse programs.

For HSQC experiments, 20–50 mg of the lignosulfonate samples was dissolved in 0.6 mL of DMSO-*d*_6_. Chemical shifts were given in parts per million, referenced to residual solvent signals (2.49 ppm for ^1^H, 39.6 ppm for ^13^C). HSQC experiments were acquired in edited mode with a relaxation delay of 0.5 s using an adiabatic pulse for the inversion of ^13^C and the GARP sequence for broadband ^13^C-decoupling, optimized for ^1^J_(CH)_ = 145 Hz. Data processing was performed with Bruker Topspin 3.1. Peak assignments were carried out according to the literature.^29−33^ Image post-processing (coloring and size) was performed with Adobe Photoshop (Adobe Systems, Inc., San José, CA) to improve clarity.

### Molecular Weight Determination

SEC was carried out on a Dionex UltiMate 3000 with an autosampler, a column oven, and a UV detector (all Thermo Fisher Scientific, Germany), coupled with an Optilab T-rEX differential refractive index (RI) detector (λ = 660 nm) and a Dawn HELEOS II MALS detector with a laser operating at 785 nm and 18 photodiodes at different measuring angles, every second of them with narrow band pass filters (±10 nm) (Wyatt Technology, Santa Barbara, CA). The analysis parameters were flow rate (0.5 mL min^–1^), column temperature (35 °C), injection volume (10 or 20 μL), UV detector at 280 nm, and RI detector at 30 °C. Separation was performed with an Agilent PLgel guard column of 7.5 × 50 mm^2^ and three Agilent PolarGel M columns of 7.5 × 300 mm^2^ (5 μm particle size) in series. DMSO with 0.5% (w/v) lithium bromide was used as the eluent. Data evaluation was performed using ASTRA software, version 7.3 (Thermo Fisher Scientific, Germany). Data processing was carried out as described by Zinovyev et al.^12^

Samples were dissolved without derivatization at room temperature in the SEC eluent (10 mg mL^–1^), shaken overnight, and filtered through a 0.45 μm PTFE syringe filter before injection. The specific refractive index increment (d*n*/d*c*)_μ_ of the lignin fractions in DMSO/LiBr (0.5% w/v) was determined using the online approach assuming 100% mass recovery of the sample and taking into consideration the accuracy of the injection system. The average (d*n*/d*c*)_μ_ of each lignin (see Table 1) was then used in the molar mass calculation.

**Table 1:** Calculated Statistical Moments for Lignosulfonate and Kraft Lignin Samples Based on SEC–MALS

		statistical moments					
no	sample	*M*_n_ [kDa]	*M*_p_ [kDa]	*M*_w_ [kDa]	*M*_z_ [kDa]	*Đ*_M_ (*M*_w_/*M*_n_)	(d*n*/d*c*)_μ_ [mL g^–1^]
1	HWLS	2.67	7.33	15.08	63.81	5.65	0.120
2	SWLS	4.19	23.53	45.57	220.32	10.88	0.110
3	HWNSSC	3.44	3.03	7.47	24.35	2.17	0.100
4	HWKL	1.56	2.35	4.11	11.00	2.63	0.150
5	SWKL	3.00	6.55	13.95	65.62	4.65	0.160

### Functional Group Analysis

#### Hydroxy and Carboxylic Acid Groups

Aliphatic hydroxy, aromatic hydroxy, and carboxylic acid group contents were determined by inverse gated ^1^H-decoupled ^31^P NMR spectroscopy. Sample preparation was adapted from the literature.^34−36^ Due to differences in solubility, different solvent mixtures were used to dissolve kraft lignins or lignosulfonates. Kraft lignins (30 mg) were dissolved in a 1:1.6 mixture of chloroform (deuterated) and pyridine (anhydrous, nondeuterated). Lignosulfonates (30 mg) were dissolved in a mixture of *N*,*N*-dimethylformamide and pyridine (anhydrous, nondeuterated; for locking and shimming, 100 μL of CDCl_3_ was added); the ratio varied between 4:1 and 5:1 to ensure optimal dissolution of the samples. For HWNSSC samples, the addition of the ionic liquid 1-ethyl-3-methylimidazolium chloride (>99%, [emim]Cl) was necessary to achieve adequate dissolution.^36^ Internal standard (4 mg of *N*-hydroxy-5-norbornene-2,3-dicarboxylic acid imide) and 0.5 mg of NMR relaxation agent, chromium (III) acetylacetonate (Cr(acac)_3_) were added along with the solvent mixture. For phosphitylation, 150 μL of 2-chloro-4,4,5,5-tetramethyl-1,3,2-dioxaphospholane was used. Spectra evaluation was carried out as described in the literature.^34−36^

#### Methoxy Groups

The methoxy group content was determined in duplicate according to Sumerskii et al.^37^ In brief, methoxy groups in the lignin sample were cleaved off by hydroiodic acid and converted into iodomethane (CH_3_I), which was then quantified by headspace GCMS.

#### Elemental Analysis

For lignosulfonates, determining sulfonic acid groups was performed indirectly as the sulfur content by elemental analysis at the Laboratory for Microanalysis Services at the University of Vienna. Prior to analysis, the samples were thoroughly dried in a vacuum oven at 40 °C and stored in an inert atmosphere. Elemental analysis was conducted as C/H/N/S analyses (oxygen was determined indirectly) on an EA 1108 CHNS-O elemental analyzer (CarloErba Instruments, CE Elantech, Inc.).^38^

### Acidic Methanolysis

Polysaccharide impurities were determined by acidic methanolysis/GCMS according to protocols in the literature.^39^ Gas chromatography/mass spectrometry (GCMS) analysis was performed on an Agilent 6890N GC and an Agilent 5975B inert XL MSD quadrupole mass selective detector (EI: 70 eV), using an Agilent HP 5MS capillary column (30 m × 0.25 mm i.d.; 0.25 μm film thickness), and helium as the carrier gas at a pressure of 0.94 bar, a flow rate of 1.1 mL min^–1^, a split flow rate of 7.5 mL min^–1^, and a split ratio of 7:1.

## Results and Discussion

### General Information on the Investigated Lignins

The technical lignins studied were subjected to extensive structural characterization to gather information on their average composition and content of functionalities.

HSQC NMR spectra of the lignins (Figure S1) showed the expected presence of syringyl units in the hardwood lignins HWLS, HWNSSC, and HWKL. SWLS showed a minor presence of syringyl units due to the proportionate use of hardwoods in pulping. The HSQC spectra of HWNSSC revealed high amounts of xylans as a result of using ultrafiltration for sample purification. In a follow-up analysis, HWNSSC was subjected to acidic methanolysis/GCMS to determine its exact content of polysaccharides, which proved to be extremely high (490 μg/mg, or 49% of the total sample mass) and consisting mostly of xylans (Figure S2). Hence, its functional group content and molar mass data should be treated with great caution. In addition to polysaccharide impurities, some samples (HWNSSC and SWKL) contained fatty acids. HWKL appeared to have undergone strong structural changes during pulping, as the assignment of native lignin structures was limited. However, newly formed tetrahydrofuranyl structures were identified (Figure S1). Assignments in the aliphatic region (top right corner) proved to be unfeasible since little to nothing has been reported in the literature about this region for lipophilic impurities in lignins.^40,41^

**Figure 1:**
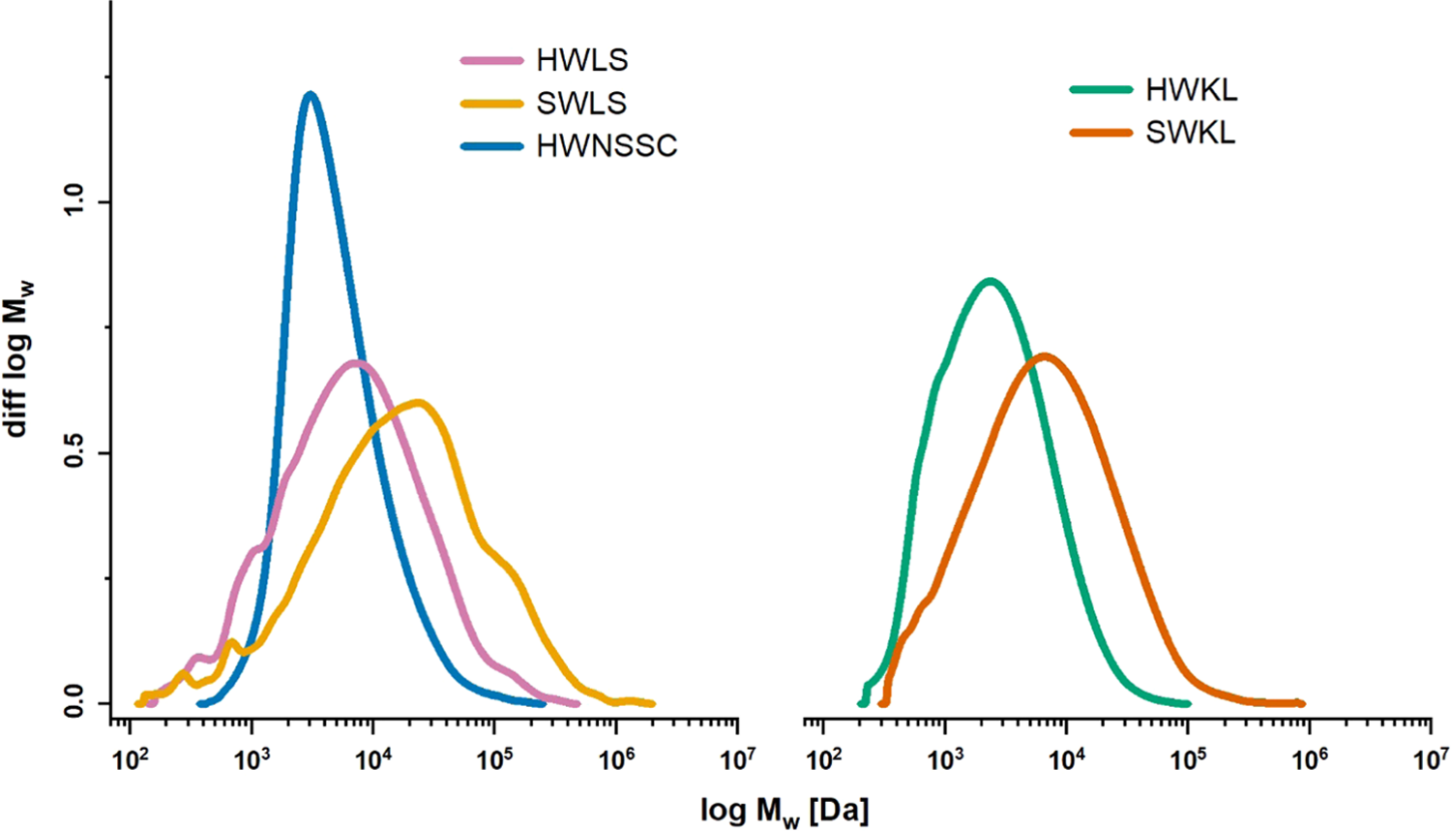
Normalized MMDs of technical lignins determined by SEC–MALS. Softwood lignins showed higher molar masses than hardwood lignins. Lignosulfonates showed higher molar masses than kraft lignins.

In general, the studied lignosulfonates showed higher M_w_ values compared to the kraft lignins, except for the NSSC lignosulfonate (i.e., HWNSSC), as expected. Moreover, the softwood lignins showed higher *M*_w_ values (13.95–45.57 kDa) than the hardwood lignins (4.11–15.08 kDa; Table 1). Also, this is in line with the literature.^42−44^ SWLS showed a very broad distribution with a dispersity (*Đ*_M_) of 10.88 and a notable shoulder in the high molar mass range above 100 kDa (Figure 1). For the other lignins, *Đ*_M_ values ranged between 2.17 and 5.65.

The methoxy (OCH_3_) group content is strongly related to the botanical origin of the lignin due to the presence of an additional OCH_3_ group per syringyl unit. Hence, hardwood lignins showed a considerably higher OCH_3_ group content (5.59–5.97 mmol/g) than softwood lignosulfonates (3.95–4.22 mmol/g; Table 2). HWNSSC showed a significantly lower OCH_3_ group content (2.31 mmol/g), which can obviously be attributed to its contamination with xylans. Naturally, lignins boast a range of functional groups, such as hydroxy and carboxylic acid groups. However, their content may change during pulping, depending on the applied process and its conditions. Thus, technical lignins show considerable differences in their functional group contents. In particular, kraft pulping is characterized by a more vigorous fragmentation compared to sulfite processes; thus, hydroxy group contents tend to deviate even more from the natural distribution. The aliphatic hydroxy group contents varied between 0.85 and 2.98 mmol/g, the aromatic hydroxy group contents between 1.83 and 4.36 mmol/g (Table 2). Carboxylic acid group contents ranged between 0.17 and 0.53 mmol/g. As stated above, functional group contents of HWNSSC should be considered with caution due to their contamination with xylans. Certainly, aliphatic hydroxy and carboxylic acid group contents are overestimated due to the abundance of xylans, whereas the aromatic hydroxyl group content is underestimated. For the lignosulfonates, the sulfonic acid (SO_3_H) group contents varied between 0.83 and 1.78 mmol/g (Table 2), which is in good agreement with the literature.^42,45^ Again, the SO_3_H group content of HWNSSC may actually be higher due to the contamination with xylans.

**Table 2:** Functional Group Contents and Relative Hydrophobicity of Lignosulfonate and Kraft Lignin Samples

		HS-GC	EA		^31^P NMR		HIC
no	sample	OCH_3_ [mmol g^–1^]	SO_3_H [mmol g^–1^]	aliph. OH [mmol g^–1^]	arom. OH [mmol g^–1^]	COOH [mmol g^–1^]	*I*_hyd_(46)
1	HWLS	5.59	1.39	1.77	2.89	0.18	0.61
2	SWLS	3.95	1.78	2.98	1.83	0.23	0.46
3	HWNSSC	2.31	0.83	4.13	1.44	0.26	0.05
4	HWKL	5.97		0.85	4.36	0.17	
5	SWKL	4.22		2.44	4.02	0.53	

For lignosulfonates, the relationship between molar mass and functionality plays an important role in their applications as surface-active agents. The relative hydrophobicity *I*_hyd_—determined by hydrophobic interaction chromatography—can be considered a suitable parameter to express this characteristic property. *I*_hyd_ is a dimensionless factor with values between 0 and 1 (i.e., low and high hydrophobicity, respectively).^47^*I*_hyd_ values of HWLS, SWLS, and HWNSSC were determined in a previous study.^46^ Interestingly, HWLS showed higher hydrophobicity (*I*_hyd_ of 0.61) than SWLS (*I*_hyd_ of 0.46) despite its lower molar mass. However, HWLS also showed a much lower SO_3_H group content, rendering it more hydrophobic.

### Efficacy of the Preparative SEC Fractionation

**Figure 2:**
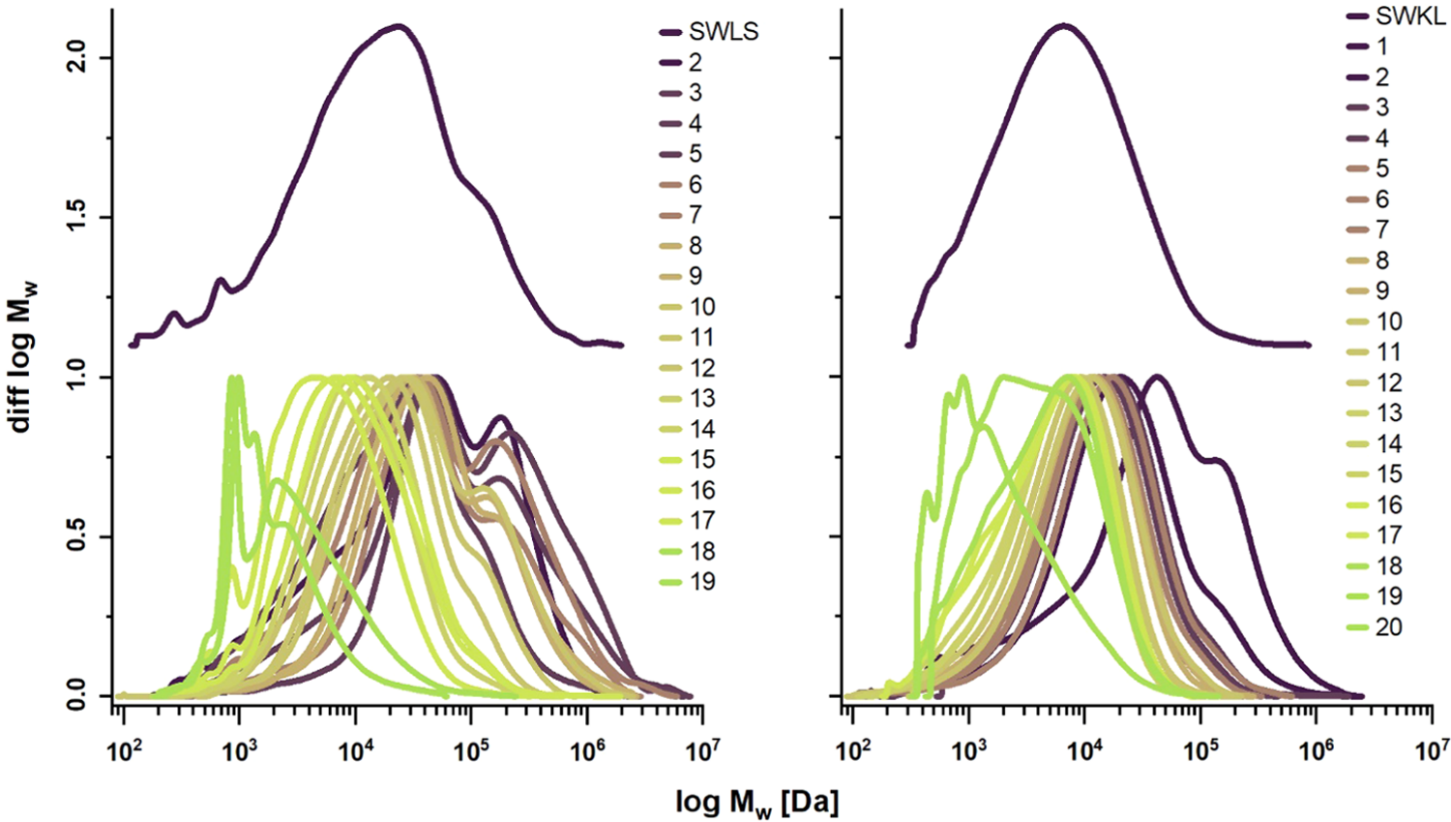
MMDs of lignin fractions (SWLS, SWKL) after preparative SEC; normalized by peak height.

**Figure 3:**
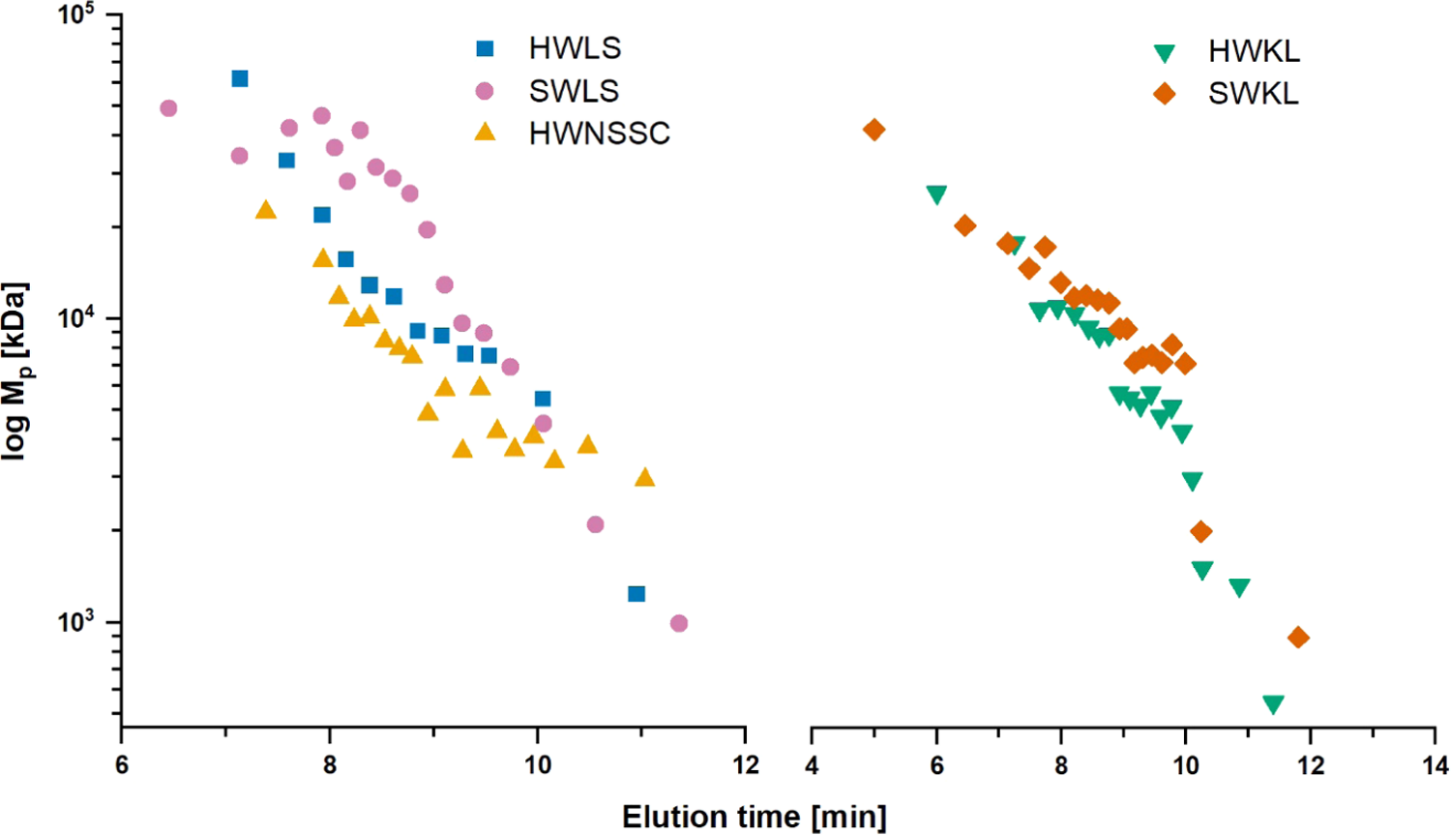
Elution time versus molar mass (*M*_p_) plots for fractionated lignins. Linearity of the plots verifies good separation efficacy by preparative SEC. SWLS shows clustering in the high molar mass range. Kraft lignins show a minor drop below 2 kDa.

Determination of the MMDs of the lignin fractions by analytical SEC was carried out to assess the separation efficacy in preparative SEC. Fractionation yielded a fine progression of (14–20) fractions with reasonably narrow MMDs (median *Đ*_M_ values of 2.6–4.1) for all lignins (Figures 2 and S3 and Table S1). In the constructed plots of elution time (in preparative SEC) versus molar mass (Figure 3), the progression of M_p_ values shows good linearity for all lignins, indicating good separation efficacy within the separation range of the preparative SEC column (1–70 kDa). However, deviations of fractions in the high molar mass range accompanied by a distinct bimodal distribution, and thus, high *Đ*_M_ values—especially for SWLS—may be related to lignin–carbohydrate complexes (LCC) present in the high molar mass range.^14^ In the low molar mass range, distortions in the fractions’ MMD also indicate a minor loss of separation efficiency.

### Functional Heterogeneity of Technical Lignins

**Figure 4:**
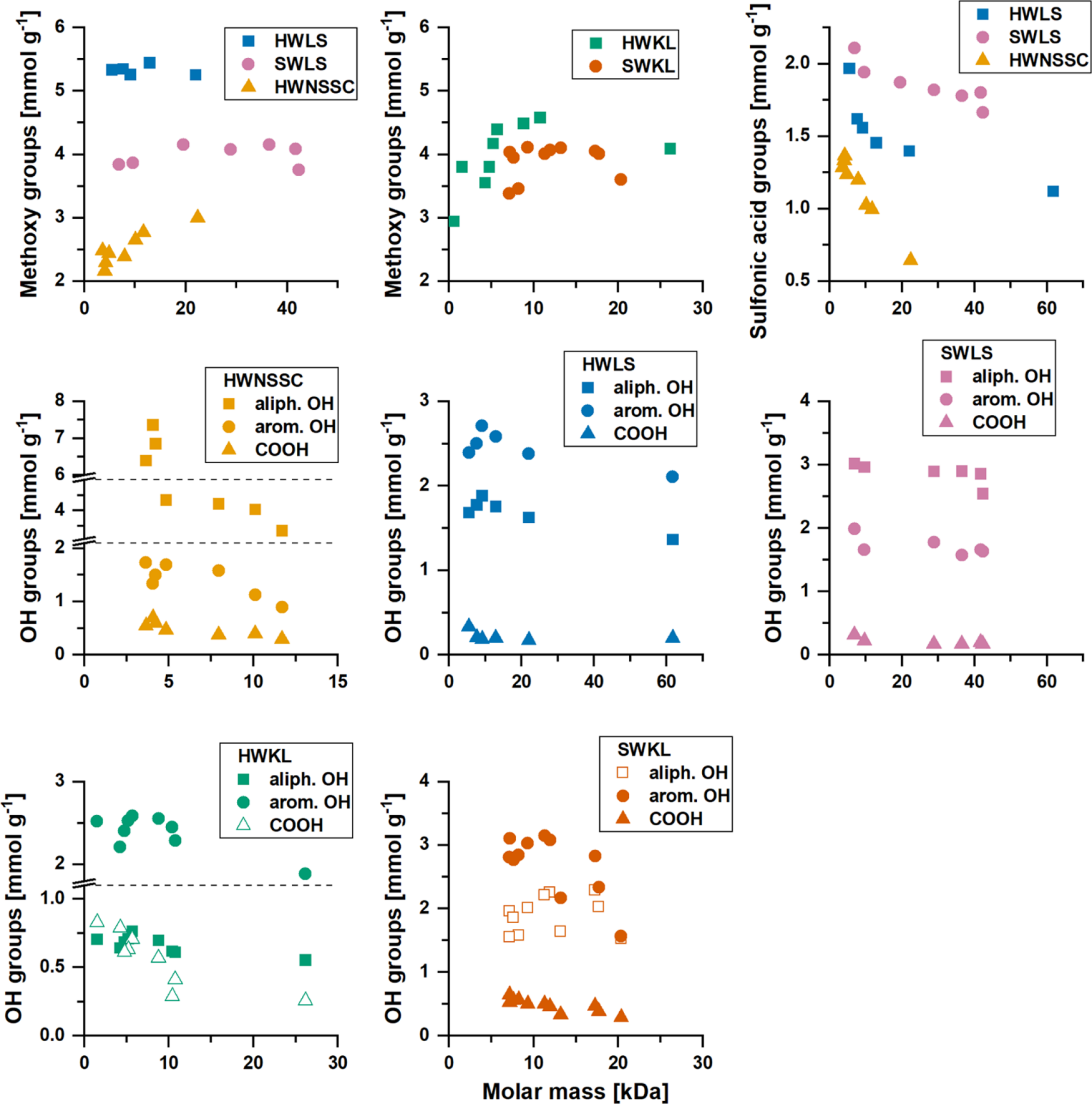
Functional group contents for technical lignins along their molar mass range. Methoxy, sulfonic acid, hydroxy, and carboxylic acid group contents are plotted versus the *M*_p_ value of their respective fraction.

Functional group contents were determined for every second lignin fraction obtained from the preparative SEC to establish characteristic profiles along the molar mass range (Figure 4 and Table S2). In general, the obtained profiles of functional heterogeneity are in line with the literature.^14,15,18,22−25,48−50^ Aliphatic hydroxy groups, especially in α-position, play a crucial role in the degradation mechanisms upon both kraft and sulfite pulping; hence, their content is prone to depletion depending on process intensity.^51,52^ In contrast, lignin fragmentation leads to an increase in the aromatic hydroxy group content due to cleavage of aryl ether bonds (i.e., β-O-4, α-O-4, and 5-O-4 bonds).^51−53^ In addition, demethoxylation is known to occur as a side reaction during alkaline pulping.^52^ For this reason, the low molar mass range showed lower contents of methoxy groups but higher contents of aromatic hydroxy and carboxylic acid (and sulfonic acid) groups, compared to its high molar mass counterpart. For lignosulfonates and HWKL, aliphatic hydroxy group contents decreased with increasing molar mass, while the opposite trend was observed for SWKL. Moreover, HWLS, SWLS, and HWKL showed a stable ratio of aliphatic to aromatic hydroxy groups (0.68, 1.70, and 0.27, respectively), while SWKL showed an increase in the ratio (from 0.55 to 0.97) with increasing molar mass. In HWNSSC, a rapid increase in the aliphatic hydroxy group content occurred below 5 kDa, indicating the presence of xylans in this molar mass range. Interestingly, the sulfonic acid group profiles exhibited different slopes for the different lignosulfonates. SWLS and HWNSSC showed a more linear relationship, while HWLS showed more of an exponential decay. This may be a reason for their differences in relative hydrophobicity. Overall, large changes in content occurred around a molar mass of 10 kDa and below, which seems to be the size threshold for degradation fragments (i.e., fragments with a change in functional group content) accumulating during pulping.

**Figure 5:**
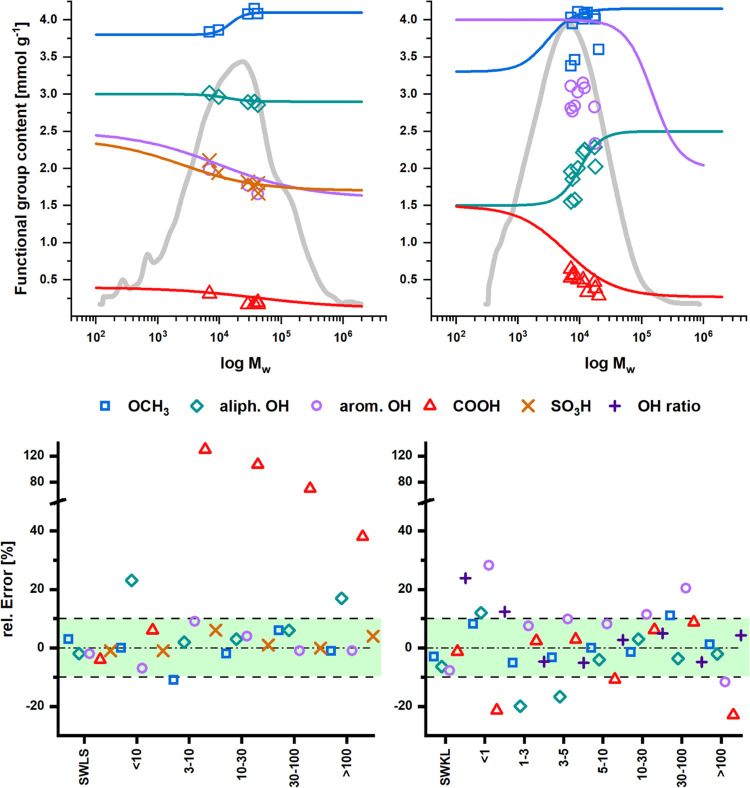
Functional heterogeneity of SWLS (top left) and SWKL (top right) used in the estimation model. Below, the optimized sigmoid functions allowed accurate estimations of the functional group contents (*F*_n_) of ultrafiltrated lignin fractions (membrane cutoff in kDa).

In general, the smooth progression of fractions allowed for better judgment of the course of the molar mass-dependent functional group contents compared to older reports in the literature. In particular, the assumption of linear trends must be questioned and should be replaced by sigmoid (S- or Z-shaped) or exponential trends to match the functional heterogeneity more accurately (Figures 5 and S5 and Table S4).

### Estimation of Functionality Based on FTD and MMD

Information on the functional heterogeneity (i.e., the molar-mass-dependent functional group contents) of a given lignin is highly valuable. Once acquired for a certain lignin, it allows the design of selective purification processes to obtain lignins with the desired properties for material usage. Currently, the design of these refining or tailoring processes relies mainly on analyses data obtained from lab-scale lignin fractionations by ultrafiltration or a sequence of solvents. Obtained in this manner, the fractions provide a rough profile of the underlying functional heterogeneity, which in turn can be used to estimate the functional group content of a targeted fraction based on its expected *M*_w_ or *M*_n_ values (Table S3 and Figure S4). Needless to say that the ability to accurately estimate the functional group contents for targeted lignin fractions can be an invaluable tool needed for meaningful process design in advance. However, estimations based on lab-scale fractionations are often compromised by their inaccurate depiction of the underlying functional heterogeneity due to the limited number of fractions involved and their typically broad MMDs; thus, they can be used only as rough guides in process design.

In our preparative SEC approach, a significantly higher number of fractions with narrower MMD can be obtained, which facilitates capturing even abrupt transitions in functional group contents along the molar mass range (Figure 4). In addition, our estimation approach involves the entire MMD in the calculation of the estimated functional group content, instead of using only statistical averages (e.g., *M*_w_ or *M*_n_). These often do not represent adequate metrics for estimations due to broad or multimodal lignin distributions. In our approach, the highly resolved profiles of functional heterogeneity are applied to a fraction’s MMD similar to a calibration function, creating the respective functionality-type distribution (FTD) from which statistical averages (i.e., *F*_n_ and *F*_w_) and the dispersity in functionality *Đ*_F_ are then calculated:
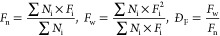
1First, the FTD of each functional group was established using a simple fit of the preparative fractions. Then, the heterogeneity profiles (i.e., fit functions) were adapted stepwise until the estimated *F*_n_ values matched closely with the measured values of the original lignin samples. As stated above, S- or Z-shaped sigmoid functions provided a much better fit than linear functions, which tended to under and overestimation at the outer margins of the distribution (Figures 5, S5, and S6)—another indication that functional group contents do not necessarily follow linear trends along the whole molar mass range. However, in some cases, the fit functions deviate to some degree from the values of the fractions to maintain a low relative error of estimation (RE) within ±10% for the functional group contents of the original lignins. In particular, the measured values from ^31^P NMR tended to show considerable differences between the fractions and the original lignins. The significant contamination of HWNSSC with xylans must also be considered when evaluating its heterogeneity profiles. Table S4 shows the final fit functions and the calculated statistical values *F*_n_, *F*_w_, and *Đ*_F_ of each FTD for all lignins. *Đ*_F_ values of the lignins were close to 1 (1.00–1.21) since their functional group contents do not change drastically (e.g., not by a factor of ≥2) with molar mass. Hence, their functionalities are, on average, rather homogeneously distributed. However, local dispersity in functionality may still pose a problem for material usage.

We also verified the accuracy of our estimation model by comparing the estimated *F*_n_ values of ultrafiltrated fractions of SWLS and SWKL, obtained in earlier studies,^14,54^ with the respective measured values (Figure 5).

**Figure 6:**
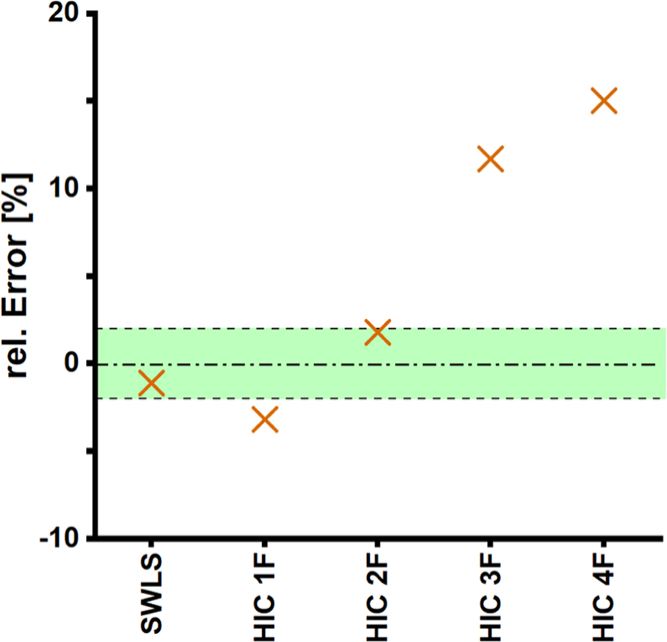
Estimation of the sulfonic acid group content of SWLS fractions after preparative HIC. High relative error of estimation (RE) indicates significant changes in the functionality composition of the fractions compared to the original SWLS.

For SWLS, the optimized fit functions showed an RE within ±4% for all estimated values. For the ultrafiltrated fractions (UF10–UF100), RE values were in median also within ±10% for most estimated values, which provided a good fit of the heterogeneity profiles. Especially the sulfonic acid and methoxy group content could be estimated consistently with high accuracy (average RE of ±2%). The hydroxy group content (i.e., aliphatic and aromatic hydroxy groups), despite some deviations for very low or very high molar mass fractions, gave an overall good fit of the profiles as well. However, the carboxylic acid group contents were consistently overestimated due to the lower content of the ultrafiltrated samples compared to SWLS and its fractions. The difference in estimation accuracy between different functional group contents could also be related to measurement inaccuracies of the reference methods, hence the reason that estimations of sulfonic acid group contents (i.e., elemental analysis) proved to be more accurate than those of hydroxy group contents (i.e., ^31^P NMR).

For SWKL, fit optimization faced some difficulties. In the case of hydroxy group contents, the optimized fits deviate to some degree from the measured values of the fractions from the preparative SEC to maintain a low RE. Accordingly, an RE within ±8% could still be achieved for all estimated values. For the ultrafiltrated fractions (F1–F7), the variation in RE was generally higher for all estimated functional group contents compared to SWLS fractions. Estimation of methoxy group contents showed good accuracy, with RE within ±10% (median of 0%). However, the hydroxy group contents showed consistently higher variation in RE values. For aliphatic hydroxy groups, the low molar mass range proved difficult to fit, while for aromatic hydroxy groups, the measured content of SWKL was higher than those of its fractions (i.e., from ultrafiltration or preparative SEC). In part, the presence of LCCs in the high molar mass range could lead to the distortions observed in FTD.^14^ In addition, kraft lignins could also exhibit naturally higher dispersity in their FTD than lignosulfonates due to the strong conversion of structures during kraft pulping, rendering their FTD more complex. This is also evident from the heterogeneity of the ratio of aliphatic to aromatic hydroxy groups in SWKL, hence estimation of this ratio was attempted for SWKL. The estimation of the aliphatic to aromatic hydroxy groups ratio showed very good accuracy for the ultrafiltrated fractions with RE within ±5% (median of +3%), although the original SWKL sample showed overestimation (+24%). Estimation of carboxylic acid group content showed also good accuracy with RE mostly within ±10% (median of +2%). Overall, a considerable improvement in estimation accuracy was achieved compared to conventional estimation approaches.

### Critical Assessment of Fractionations and Separations

Basically, the acquired profiles of functional heterogeneity indicate the average functionality composition (i.e., average functional group content) of a lignin along its molar mass range. However, local dispersity in functionality at a certain molar mass cannot simply be ruled out. In fact, multidimensional separations based on molar mass and functionality reveal exactly this polydisperse character of lignins.^46,49,54^ The development of selective separation processes based on functionality is usually tedious since the effect of separation is often obscured by a molar mass-dependent shift in the functionality-type distribution (FTD). However, the application of our estimation tool allowed us to determine any change in a fraction’s FTD independent of molar mass. Selective separations based on functionality aim at enriching fractions with species of higher or lower functionality, which leads to the favored shift in their average functionality composition. Hence, their determined functional group contents deviate, to a certain extent, from their estimated counterparts. In this way, the separation effect can be assessed more thoroughly than previously possible.

We verified the applicability of our assessment approach on SWLS fractions obtained after preparative HIC (Table S5 and Figure 6).^54^ Comparison of the measured sulfonic acid group contents with the estimated values led generally to the same conclusion—a significant impact of sulfonic acid group content on the separation—but at the same time provided more details on the extent of the change in their FTD; +3.2, −1.8, −11.7, and −15.0 (±2)% in the sulfonic acid group content for 1F, 2F, 3F, and 4F, respectively.

## Conclusions

Aqueous SEC proved to be a reliable and versatile system for the preparative fractionation of both kraft lignins and lignosulfonates, according to molar mass. Moreover, preparative SEC of lignins permitted offline characterization of narrow molar mass segments and thus the determination of individual functional heterogeneities (i.e., the molar-mass-dependent functional group profiles) of technical lignins. Considerable differences in the shape of these profiles were observed for the different investigated technical lignins. In contrast to previous assumptions, the shape of these profiles followed sigmoid or exponential trends rather than linear ones. Based on the highly resolved profiles of functional heterogeneity, we also determined the dispersity in functionality *Đ*_F_ of lignins via their functionality-type distributions (FTD). *Đ*_F_ values of 1.00–1.21 indicated, on average, a homogeneous distribution of their functionalities. In addition, a robust calculation approach was developed for the estimation of the functional group contents of lignin fractions simply based on their FTD and MMD. Thus, we propose a valuable calculation tool, which adequately meets the critical demands for accurate estimations and can be used to simulate the effects on functional group content of changes in the MMD due to applied or intended purification processes. In addition, the calculation tool enables the evaluation of separations based on functionality more thoroughly regarding their separation efficiency than was hitherto possible.

## Supporting Information Available

2D NMR, hemicellulose composition, MMDs after preparative SEC, molar mass data, functional group data, linear estimation models, functional heterogeneity profiles, and FTD characteristics (PDF)
