# Supplementary material for: High-Resolution Profiling of the Functional Heterogeneity of Technical Lignins
Source: Biomacromolecules. 2022 Feb 25;23(3):1413–22. doi: 10.1021/acs.biomac.1c01630 (PMC8924861; doi:10.1021/acs.biomac.1c01630)
Supplement: Supplementary file 1 — bm1c01630_si_001.pdf [file bm1c01630_si_001.pdf]

High-Resolution Profiling of the Functional Heterogeneity of Technical Lignins

Supporting Information

Oliver Musl,[1] Samira Galler,[1] Gerhild Wurzer,[1] Markus Bacher,[1] Irina Sulaeva,[2] Ivan Sumerskii,[2] Arnulf Kai Mahler, [3] Thomas Rosenau,[1] and Antje Potthast[1*]

* Correspondence: antje.potthast@boku.ac.at; Tel.: +43 1 47654 77412

Number of pages: 15; Number of figures: 6; Number of tables: 5

Table of contents

Page S1-3	**Figure S1**. HSQC NMR spectra of the lignin samples.
Page S3	**Figure S2.** Hemicellulose composition of HWNSSC.
Page S4–5	**Figure S3.** MMDs of lignin fractions after preparative SEC.
Page S5–7	**Table S1**. Molar mass data on lignin fractions from preparative SEC.
Page S8–9	**Table S2**. Functional group contents of lignin fractions after preparative SEC.
Page S9	**Table S3**. Functional group contents and M_w values of SWLS (UF10–100) and SWKL (F1–7) fractions after ultrafiltration.
Page S10	**Figure S4**. Estimation of functional group contents of SWLS and SWKL fractions based on their M_w values using linear fits.
Page S11	**Figure S5.** Functional heterogeneity profiles of HWLS, HWNSSC and HWKL.
Page S12–13	**Table S4.** Fitting functions of the heterogeneity profiles and the respective FTD characteristics of the lignins.
Page S14	**Figure S6**. Estimation of functional group contents of ultrafiltrated SWLS and SWKL fractions based on their MMD using linear fits.
Page S15	**Table S5**. Data on SWLS fractions after preparative HIC.

Figure S1. HSQC NMR spectra of the used lignosulfonate and kraft lignin samples. Selected structural motifs are highlighted: ▪ $S_{2,6/2',6'}$ (S unit/α-oxo-S unit); ▪ $G_{2,5,6}$ (G unit); ▪ $H_{2,6}$ (H unit); ▪ $=CH_2$; ▪ fa (fatty acids); ▪ $A_{\alpha,\beta,\gamma}$ (β-aryl ether); ▪ $B_{\alpha,\beta,\gamma}$ (phenylcoumaran); ▪ $C_{\alpha,\beta,\gamma}$ (resinol); ▪ OCH_3 (methoxy group); ▪ $\alpha SA_{\alpha,\beta,\gamma}$ (sulfo group in α-position); ▪ xyl (xylans); ▪ $T_{\alpha,\beta,\gamma}$ (tetrahydrofuranyl).

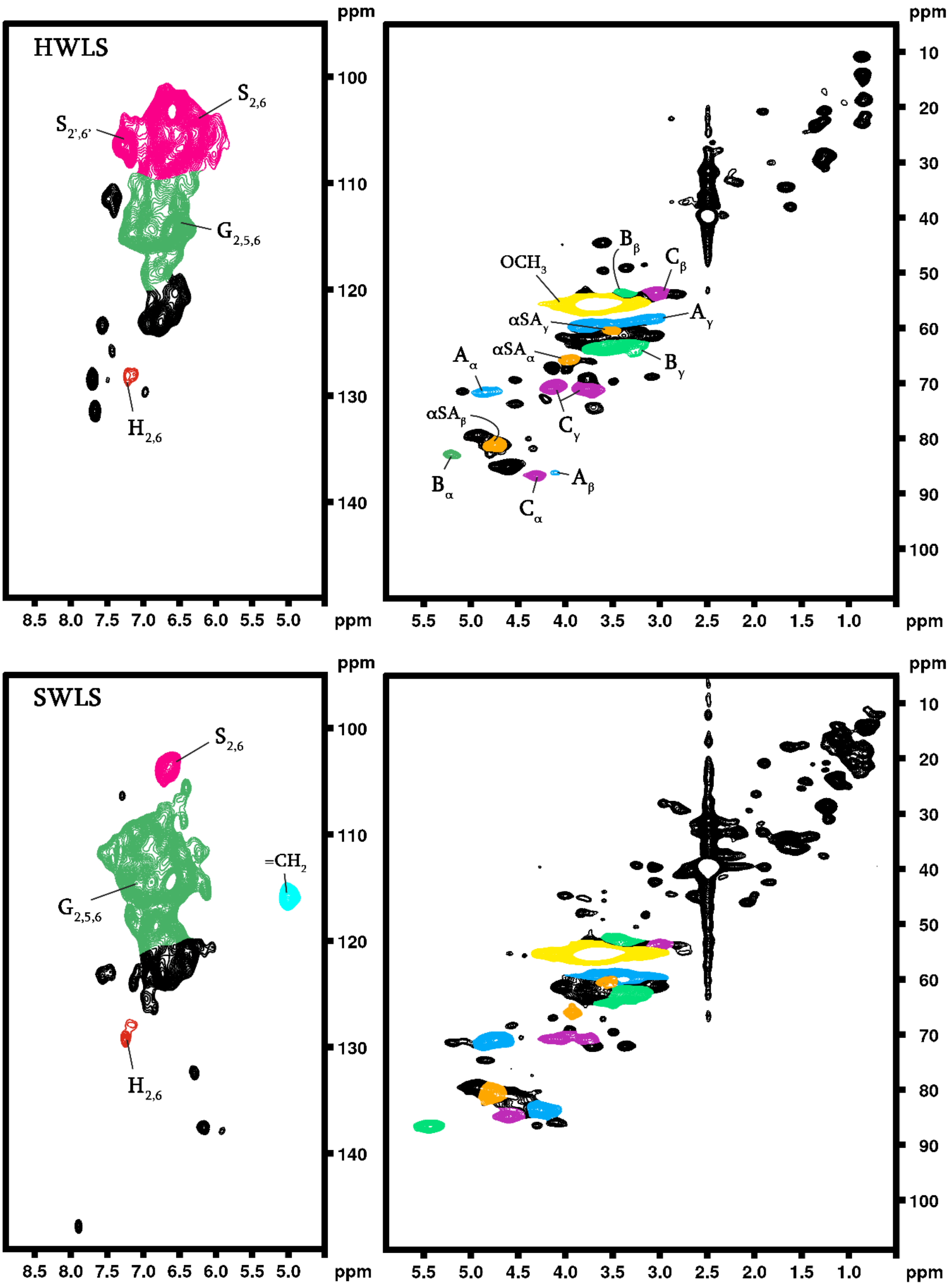

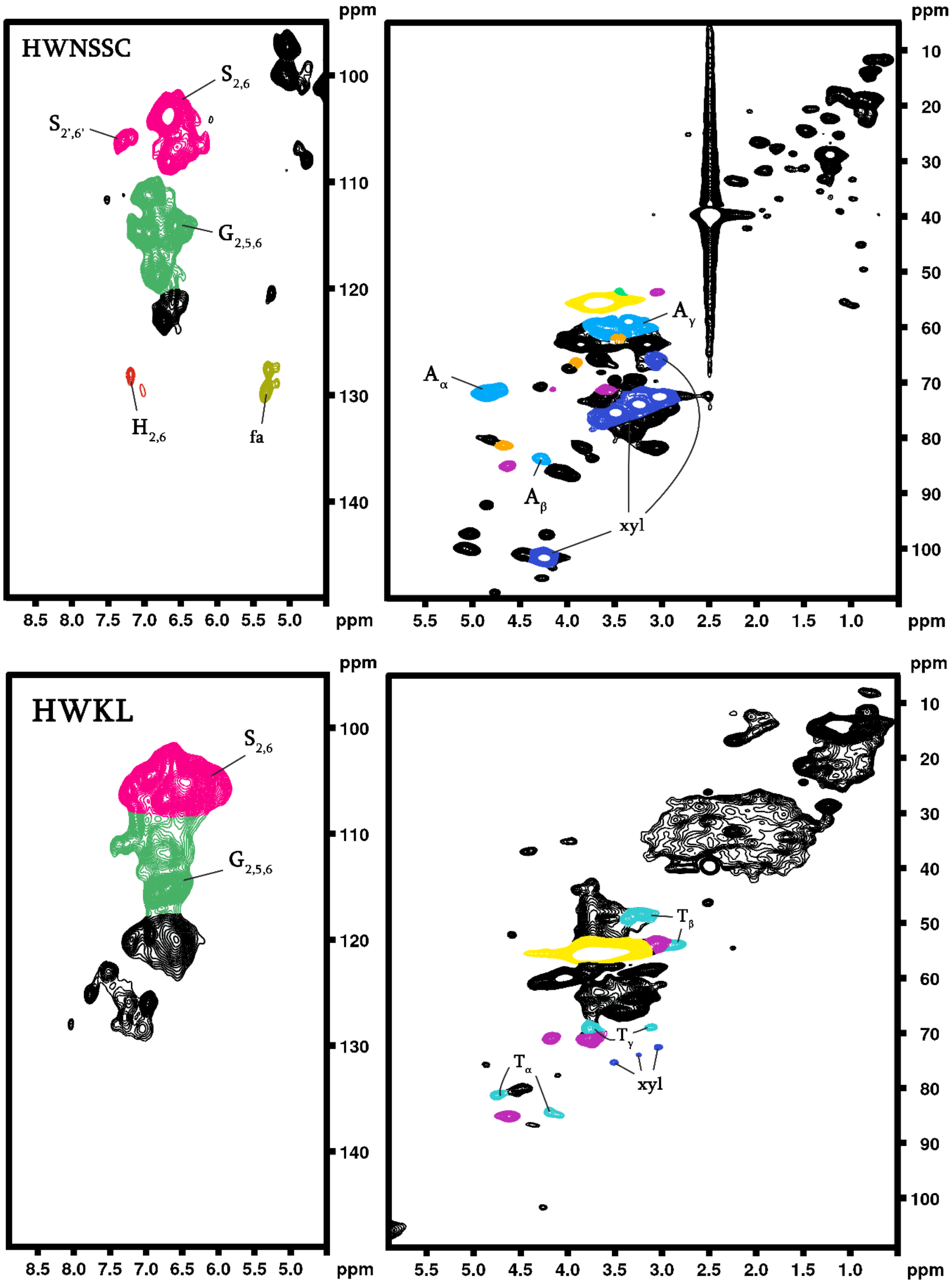
HWNSSC
S2,6
S2',6'
G2,5,6
H2,6
fa
Aα
Aβ
Aγ
xyl
HWKL
S2,6
G2,5,6
Tα
Tβ
Tγ
xyl
ppm

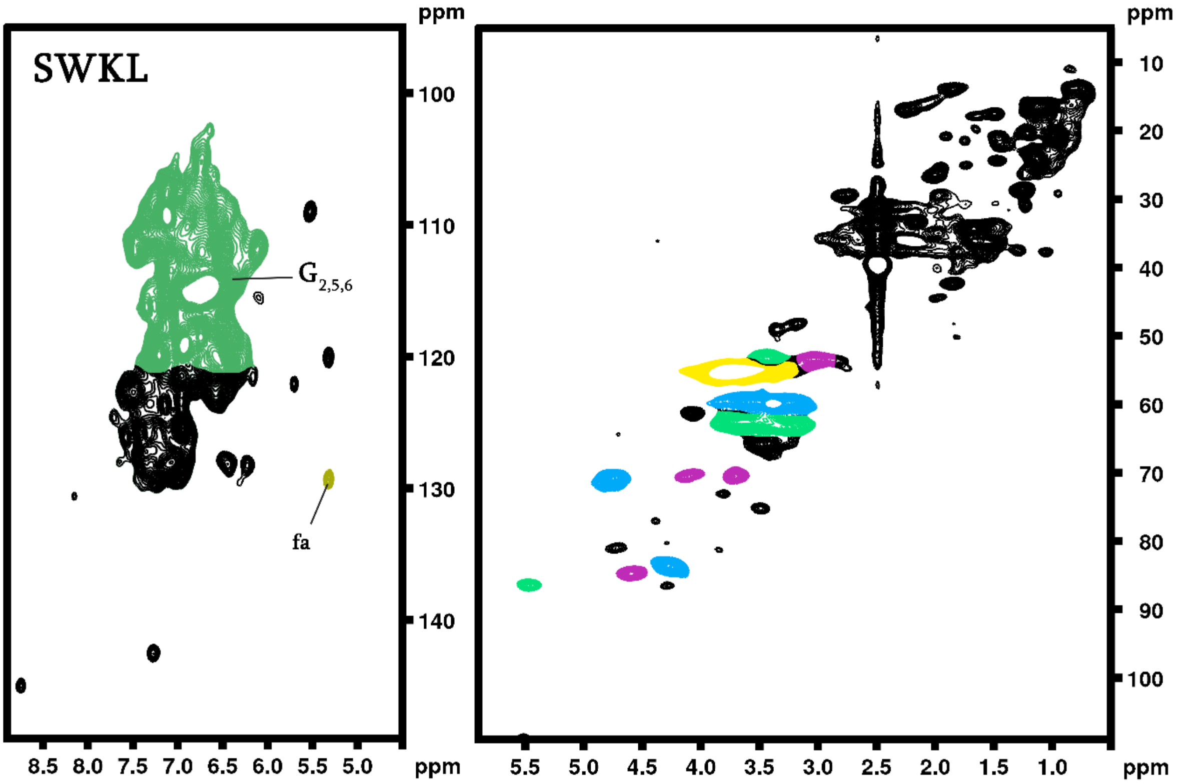

Figure S2. Hemicellulose composition of ultrafiltrated HWNSSC lignosulfonate revealed high amounts of xylans. Determined sugar monomer contents after acid methanolysis: arabinose (Ara), rhamnose (Rha), fucose (Fuc), xylose (Xyl), galacturonic acid (GalUA), 4-O-methylglucuronic acid (4OMeGlcUA), mannose (Man), galactose (Gal), glucose (Glc), and glucuronic acid (GlcUA).

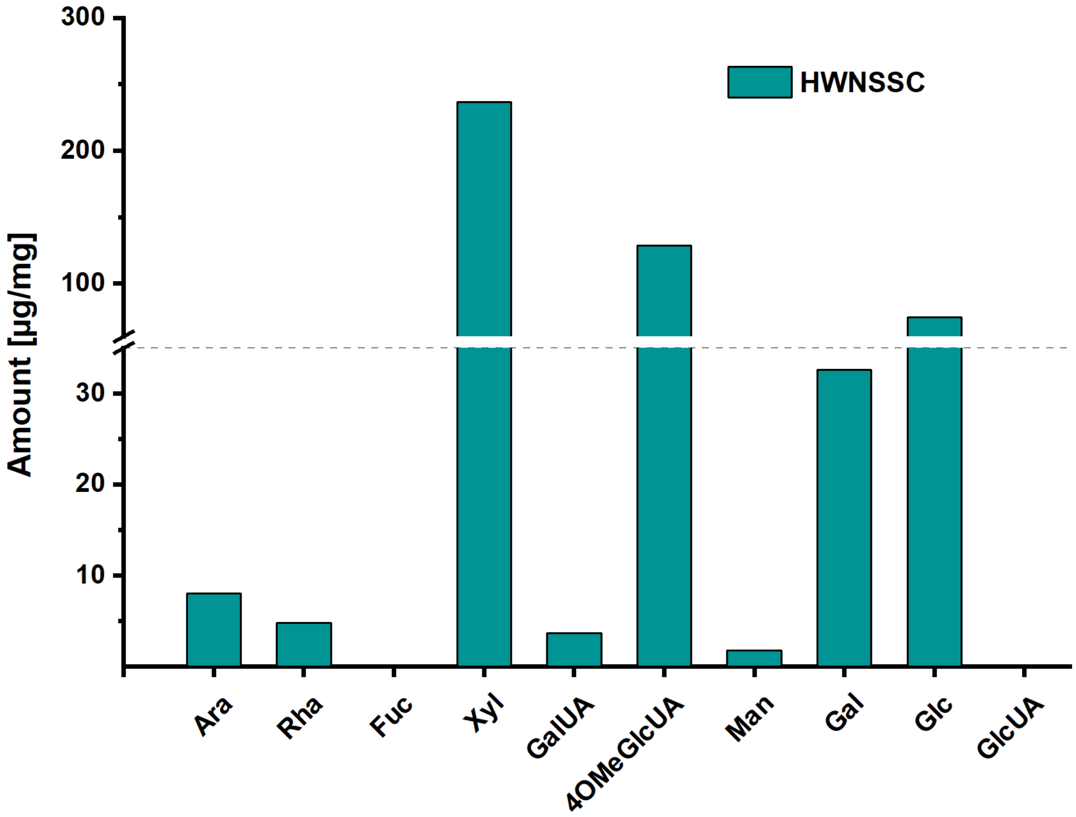

Figure S3. MMDs of lignin fractions after preparative SEC; normalized by peak height.

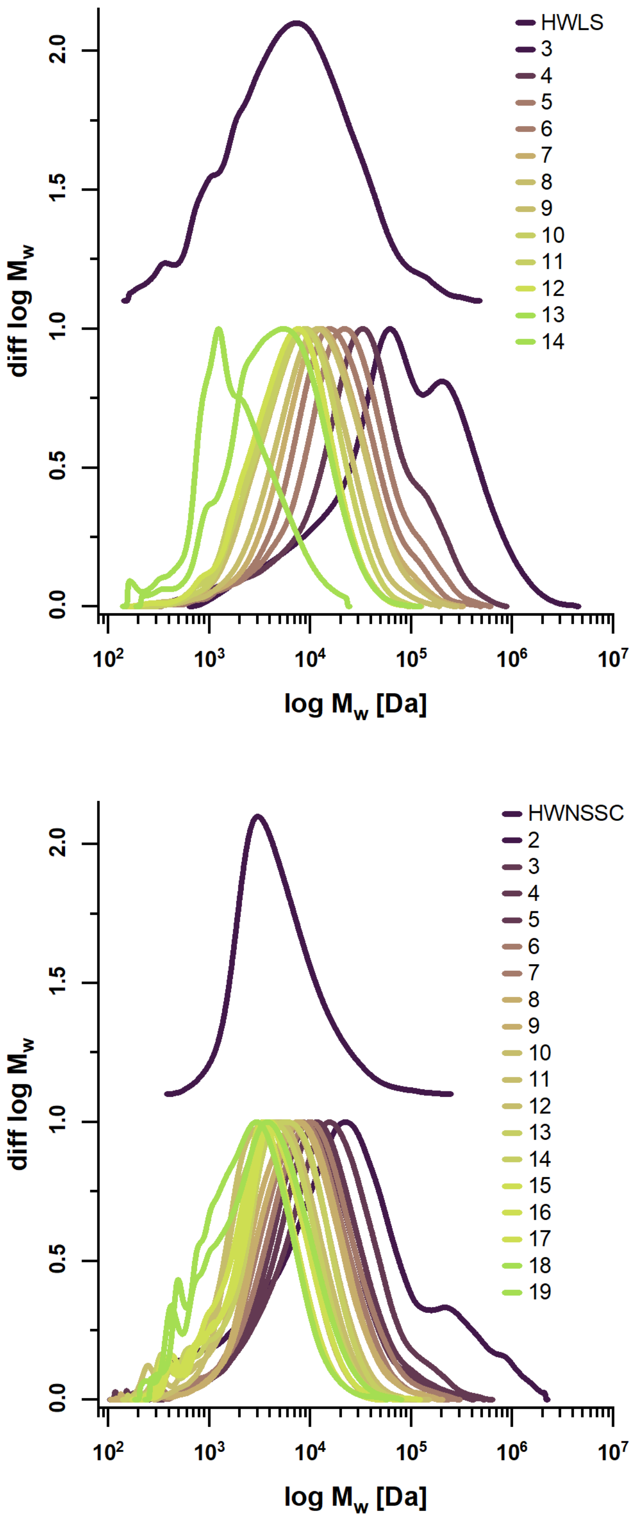

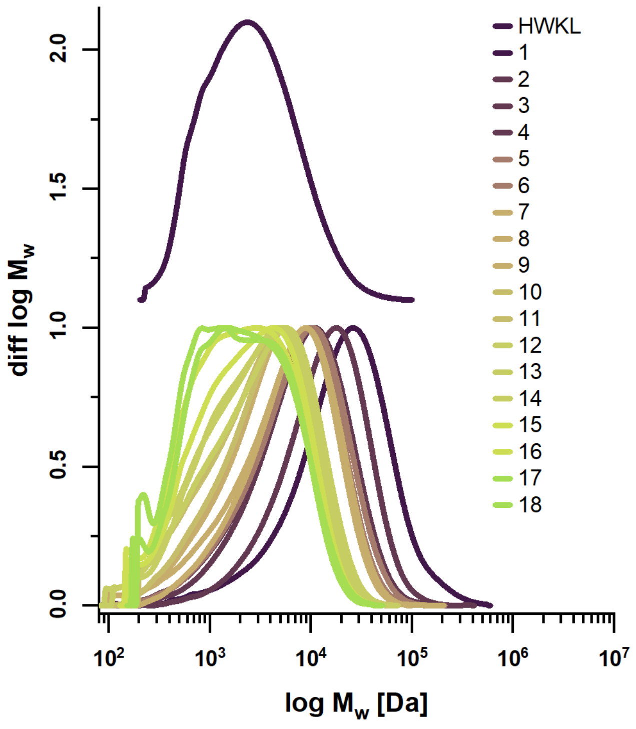

Table S1. Molar mass data on lignin fractions from preparative SEC.

Sample	Statistical moments M_n kDa	M_p kDa	M_w kDa	M_z kDa	Đ (Mw/Mn)
**HWLS**	2.67	7.33	15.08	63.81	5.65
**3**	22.83	61.68	188.74	640.20	8.27
**4**	11.19	33.17	58.68	157.89	5.24
**5**	11.14	21.98	37.88	100.80	3.40
**6**	8.81	15.70	27.72	71.57	3.15
**7**	7.23	12.89	21.28	50.80	2.94
**8**	7.06	11.83	18.92	43.73	2.68
**9**	5.18	9.10	13.27	30.05	2.56
**10**	4.92	8.80	10.96	20.90	2.23
**11**	4.18	7.66	8.97	16.77	2.15
**12**	4.01	7.55	8.89	16.68	2.22
**13**	2.73	5.45	7.30	15.89	2.67
**14**	1.26	1.24	2.79	6.00	2.21

Sample	Statistical moments<br>M_n<br>kDa	M_p<br>kDa	M_w<br>kDa	M_z<br>kDa	Đ<br>(Mw/Mn)
**SWLS**	4.19	23.53	45.57	220.32	10.88
**2**	6.99	49.21	101.30	307.17	14.49
**3**	6.34	34.28	57.27	312.95	9.03
**4**	10.49	42.31	214.17	1171.08	20.42
**5**	24.13	46.30	287.41	1180.78	11.91
**6**	19.12	36.50	191.10	880.43	9.99
**7**	10.70	28.24	120.46	573.18	11.26
**8**	16.91	41.72	97.37	348.35	5.76
**9**	18.23	31.48	112.16	490.99	6.15
**10**	14.52	28.82	92.85	375.67	6.39
**11**	11.61	25.69	47.18	143.37	4.06
**12**	8.00	19.56	30.37	86.52	3.80
**13**	6.72	12.89	21.42	63.57	3.19
**14**	5.48	9.64	16.68	51.19	3.04
**15**	5.42	8.96	21.60	107.97	3.99
**16**	4.38	6.90	17.41	64.38	3.97
**17**	2.97	4.51	9.59	30.52	3.23
**18**	1.70	2.09	5.70	27.43	3.35
**19**	1.47	0.99	3.18	10.34	2.16

Sample	Statistical moments<br>M_n<br>kDa	M_p<br>kDa	M_w<br>kDa	M_z<br>kDa	Đ<br>(Mw/Mn)
**HWNSSC**	3.44	3.03	7.47	24.35	2.17
**2**	4.86	22.38	93.36	583.56	19.21
**3**	6.26	15.54	27.94	91.89	4.46
**4**	5.93	11.70	20.43	63.50	3.45
**5**	5.49	9.86	18.20	63.32	3.32
**6**	5.19	10.10	16.18	44.38	3.12
**7**	4.96	8.42	13.11	31.42	2.64
**8**	4.62	7.95	12.19	28.79	2.64
**9**	4.54	7.48	11.67	28.61	2.57
**10**	3.11	4.84	6.91	14.83	2.22
**11**	3.03	5.83	8.12	16.61	2.68
**12**	2.02	3.66	5.05	10.40	2.50
**13**	2.90	5.87	7.80	15.06	2.69
**14**	2.35	4.23	6.35	13.59	2.70
**15**	2.28	3.69	5.57	12.40	2.44
**16**	2.52	4.07	5.61	10.58	2.23
**17**	2.17	3.36	4.43	8.01	2.04
**18**	1.80	3.78	5.23	12.25	2.91
**19**	1.60	2.95	3.63	8.07	2.27

Sample	Statistical moments				
	M_n kDa	M_p kDa	M_w kDa	M_z kDa	Đ (Mw/Mn)
**HWKL**	1.56	2.35	4.11	11.00	2.63
**1**	8.99	26.15	34.16	86.11	3.80
**2**	7.31	17.81	19.66	38.66	2.69
**3**	4.17	10.75	12.35	25.99	2.96
**4**	3.60	10.93	12.31	25.52	3.42
**5**	3.53	10.43	11.57	23.52	3.28
**6**	3.46	9.39	10.25	20.56	2.96
**7**	3.38	8.79	9.29	18.23	2.75
**8**	2.22	8.90	9.10	18.24	4.10
**9**	2.04	5.69	5.82	10.90	2.85
**10**	1.95	5.47	5.63	10.85	2.89
**11**	1.63	5.20	5.32	11.23	3.26
**12**	1.37	5.69	5.39	11.47	3.93
**13**	1.32	4.77	4.53	9.75	3.43
**14**	1.53	5.14	4.88	10.37	3.19
**15**	1.29	4.26	4.07	8.69	3.16
**16**	1.12	2.97	3.57	8.09	3.19
**17**	1.15	1.52	3.53	8.22	3.07
**18**	1.07	1.33	3.27	7.49	3.06

Sample	Statistical moments				
	M_n kDa	M_p kDa	M_w kDa	M_z kDa	Đ (Mw/Mn)
**SWKL**	3.00	6.55	13.95	65.62	4.65
**1**	7.33	42.22	98.93	347.36	13.50
**2**	8.13	20.34	46.62	212.98	5.73
**3**	7.42	17.72	29.53	103.27	3.98
**4**	7.35	14.73	23.05	61.25	3.14
**5**	7.08	17.28	28.22	84.63	3.99
**6**	5.28	13.19	20.03	51.35	3.79
**7**	4.74	11.74	15.73	34.84	3.32
**8**	4.44	11.94	15.55	35.21	3.50
**9**	4.29	11.54	14.14	31.15	3.30
**10**	4.67	11.31	15.73	36.91	3.37
**11**	3.60	9.28	10.85	22.32	3.01
**12**	3.42	9.30	10.70	21.94	3.13
**13**	3.11	7.18	8.10	15.29	2.60
**14**	3.01	7.47	8.31	16.16	2.76
**15**	3.08	7.62	8.64	17.96	2.81
**16**	2.68	7.20	8.08	16.97	3.01
**17**	2.50	8.23	8.08	16.61	3.23
**18**	2.48	7.15	7.24	15.82	2.92
**19**	2.20	2.00	6.31	15.90	2.87
**20**	1.19	0.90	3.52	13.32	2.96

Table S2. Functional group contents (i.e., methoxy, hydroxy, carboxylic acid, and sulfonic acid group contents) of lignosulfonate and kraft lignin fractions after preparative SEC.

Nr.	Sample	HS-GC OCH_3 mmol g^{-1}	EA SO_3H mmol g^{-1}	^{31}P NMR aliph. OH mmol g^{-1}	arom. OH mmol g^{-1}	COOH mmol g^{-1}
0	HWLS	5.59	1.39	1.77	2.89	0.18
1	F#3	3.87	1.12	1.37	2.10	0.20
2	F#5	5.26	1.40	1.62	2.38	0.18
3	F#7	5.44	1.46	1.76	2.59	0.20
4	F#9	5.26	1.56	1.88	2.71	0.18
5	F#11	5.35	1.62	1.77	2.50	0.20
6	F#13	5.34	1.97	1.68	2.39	0.33

Nr.	Sample	HS-GC OCH_3 mmol g^{-1}	EA SO_3H mmol g^{-1}	^{31}P NMR aliph. OH mmol g^{-1}	arom. OH mmol g^{-1}	COOH mmol g^{-1}
0	SWLS	3.95	1.78	2.98	1.83	0.23
1	F#4	3.75	1.66	2.54	1.63	0.17
2	F#6	4.15	1.78	2.90	1.57	0.17
3	F#8	4.08	1.80	2.86	1.66	0.20
4	F#10	4.08	1.82	2.89	1.77	0.17
5	F#12	4.15	1.87	4.54	2.41	0.58
6	F#14	3.86	1.94	2.96	1.66	0.22
7	F#16	3.84	2.11	3.02	1.99	0.31

Nr.	Sample	HS-GC OCH_3 mmol g^{-1}	EA SO_3H mmol g^{-1}	^{31}P NMR aliph. OH mmol g^{-1}	arom. OH mmol g^{-1}	COOH mmol g^{-1}
0	HWNSSC	2.31	0.83	4.13	1.44	0.26
1	F#2	3.00	0.64	3.35	0.90	0.29
2	F#4	2.77	0.99	4.04	1.13	0.39
3	F#6	2.65	1.03	4.22	1.58	0.38
4	F#8	2.39	1.20	4.34	1.69	0.47
5	F#10	2.44	1.24	6.39	1.73	0.54
6	F#12	2.48	1.29	6.86	1.50	0.60
7	F#14	2.29	1.37	7.36	1.34	0.70
8	F#16	4.05	1.34	-	-	-

Nr.	Sample	HS-GC OCH_3 mmol g^{-1}	^{31}P NMR aliph. OH mmol g^{-1}	arom. OH mmol g^{-1}	COOH mmol g^{-1}
0	HWKL	5.97	0.85	4.36	0.17
1	F#1	4.09	0.55	1.88	0.26
2	F#3	4.58	0.61	2.29	0.41
3	F#5	3.52	0.62	2.45	0.29
4	F#7	4.49	0.70	2.55	0.57
5	F#9	4.40	0.76	2.59	0.70
6	F#11	4.17	0.71	2.53	0.63
7	F#13	3.80	0.68	2.41	0.61
8	F#15	3.55	0.64	2.21	0.79
9	F#17	3.80	0.7	2.52	0.83
10	F#19	2.94	-	-	-

Nr.	Sample	HS-GC<br>OCH_3<br>mmol g^{-1}	^{31}P NMR<br>aliph. OH<br>mmol g^{-1}	 arom. OH<br>mmol g^{-1}	 COOH<br>mmol g^{-1}
**0**	SWKL	4.22	1.80	3.15	0.37
**1**	F#2	3.60	1.52	1.56	0.29
**2**	F#3	4.01	2.03	2.33	0.39
**3**	F#5	4.05	2.29	2.83	0.47
**4**	F#6	4.10	1.64	2.17	0.34
**5**	F#8	4.07	2.25	3.08	0.46
**6**	F#10	4.01	2.22	3.15	0.50
**7**	F#12	4.11	2.01	3.03	0.5
**8**	F#13	4.03	1.96	3.11	0.53
**9**	F#15	3.95	1.86	2.77	0.56
**10**	F#17	3.46	1.58	2.84	0.57
**11**	F#18	3.38	1.55	2.81	0.64

Table S3. Functional group contents and M_w values of SWLS (UF10–100) and SWKL (F1–7) fractions after ultrafiltration.[1,2]

Nr.	Sample	SEC-MALS<br>Mw<br>kDa	HS-GC<br>OCH_3<br>mmol g^{-1}	EA<br>SO_3H<br>mmol g^{-1}	^{31}P NMR<br>aliph. OH<br>mmol g^{-1}	 arom. OH<br>mmol g^{-1}	 COOH<br>mmol g^{-1}
**1**	UF10	3.57	3.83	2.01	2.43	2.31	0.32
**2**	UF3-10	5.19	4.30	1.83	2.93	1.94	0.14
**3**	UF10-30	23.30	4.13	1.79	2.84	1.81	0.13
**4**	UF30-100	72.09	3.87	1.74	2.75	1.78	0.12
**5**	UF100	85.68	4.13	1.66	2.49	1.72	0.13

Nr.	Sample	Cutoff<br>kDa	SEC-MALS<br>Mw<br>kDa	HS-GC<br>OCH_3<br>mmol g^{-1}	^{31}P NMR<br>aliph. OH<br>mmol g^{-1}	 arom. OH<br>mmol g^{-1}	 COOH<br>mmol g^{-1}
**1**	F1	>100	141.10	4.10	2.54	2.72	0.39
**2**	F2	30-100	15.83	3.70	2.35	3.26	0.51
**3**	F3	10-30	8.33	4.10	1.92	3.58	0.70
**4**	F4	5-10	6.73	4.00	1.95	3.69	0.91
**5**	F5	3-5	2.83	3.90	1.98	3.64	1.01
**6**	F6	1-3	2.32	3.90	1.99	3.72	1.08
**7**	F7	<1	2.23	3.40	1.41	3.12	1.43

Figure S4. Estimation of functional group contents of SWLS and SWKL fractions after preparative SEC based on their Mw values. Linear fits are based on the ultrafiltrated UF10–UF100 and F1–F7 fractions, respectively.

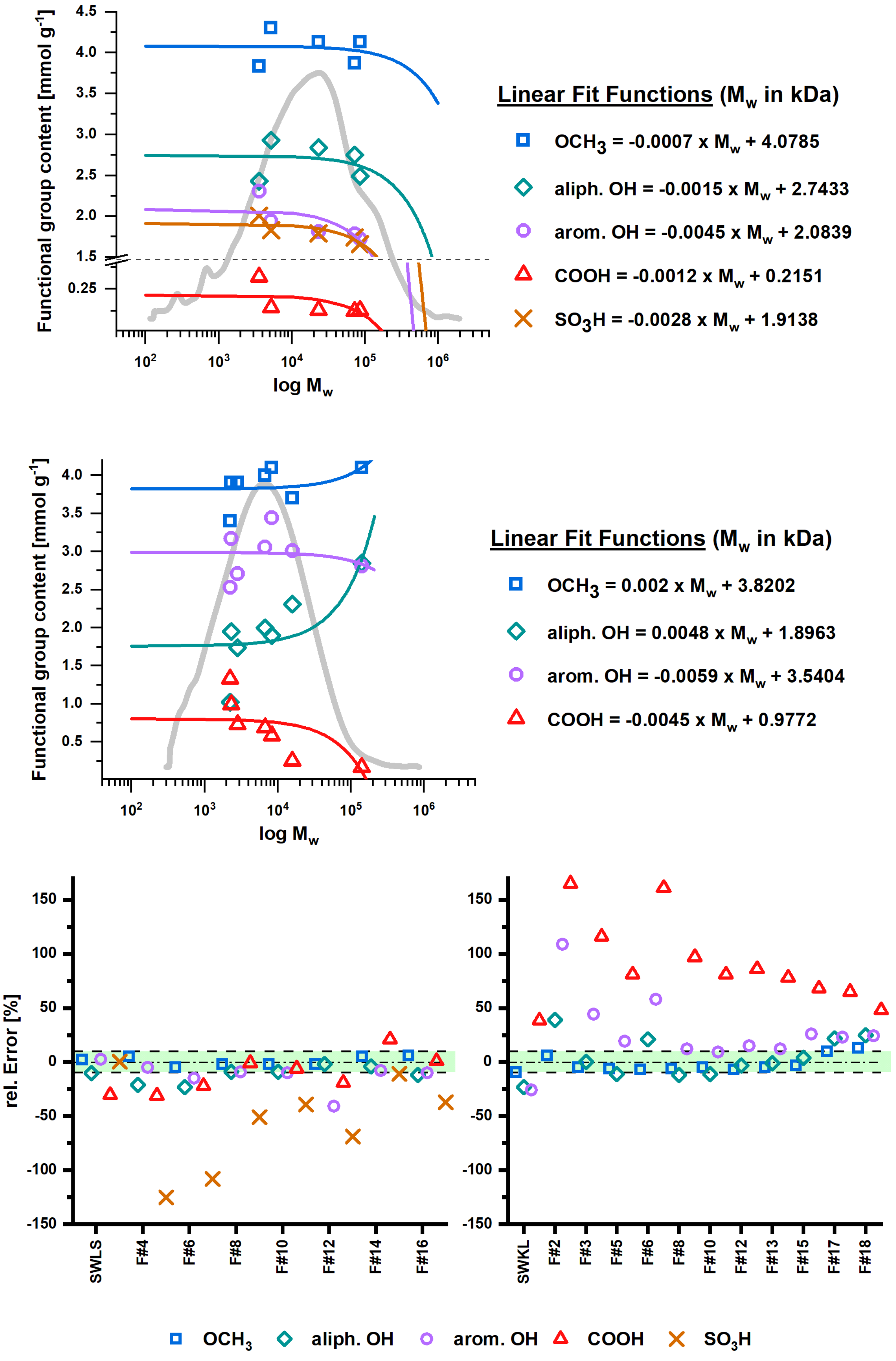

Figure S5. Functional heterogeneity profiles of HWLS, HWNSSC and HWKL. MMD of the respective lignin is in the background.

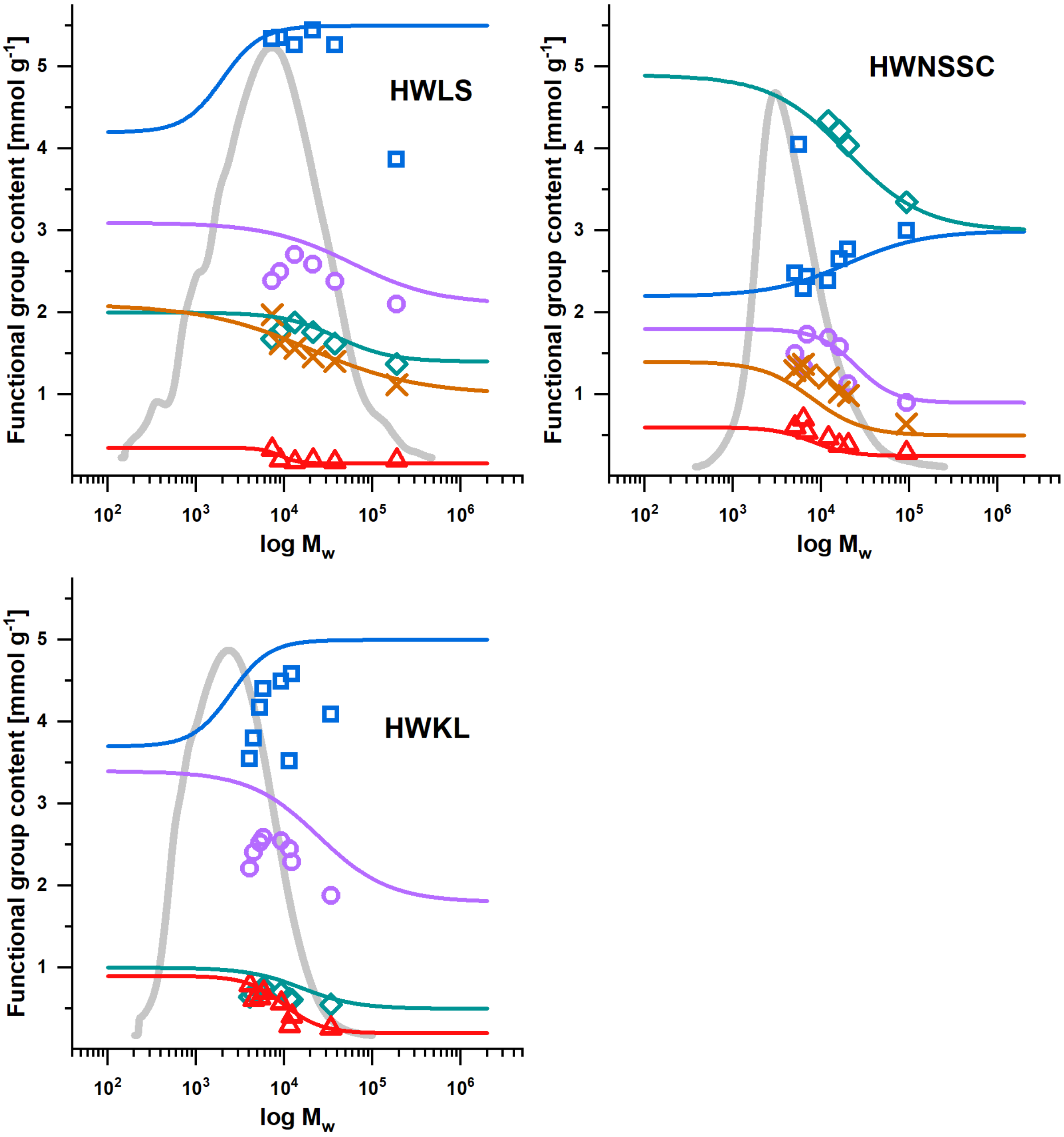

Table S4. Fitting functions of the heterogeneity profiles and the respective FTD characteristics for the lignin samples. Relative error of estimation (RE) is calculated based on the measured values.

Sample	Functionality	Fit	RE	Statistical moments of the FTD		
				F_n mmol g^{-1}	F_w mmol g^{-1}	$Đ_F$
HWLS	OCH_3	$\frac{5.5+(4.2-5.5)}{\left[1+\left(\frac{M_w}{2\,000}\right)^{2.0}\right]}$	-2%	5.45	5.46	1.00
	aliph.OH	$\frac{1.4+(2.0-1.4)}{\left[1+\left(\frac{M_w}{4\,000}\right)^{1.4}\right]}$	-3%	1.72	1.74	1.01
	arom. OH	$\frac{2.1+(3.1-2.1)}{\left[1+\left(\frac{M_w}{60\,000}\right)^{0.9}\right]}$	-7%	2.70	2.72	1.01
	COOH	$\frac{0.16+(0.35-0.16)}{\left[1+\left(\frac{M_w}{9\,000}\right)^{4.0}\right]}$	+5%	0.19	0.21	1.11
	SO_3H	$\frac{1.0+(2.1-1.0)}{\left[1+\left(\frac{M_w}{20\,000}\right)^{0.7}\right]}$	+5%	1.46	1.49	1.02
SWLS	OCH_3	$\frac{4.1+(3.8-4.1)}{\left[1+\left(\frac{M_w}{15\,000}\right)^{3.7}\right]}$	+3%	4.08	4.08	1.00
	aliph.OH	$\frac{2.9+(3.0-2.9)}{\left[1+\left(\frac{M_w}{13\,000}\right)^{2.4}\right]}$	-2%	2.91	2.91	1.00
	arom. OH	$\frac{1.6+(2.5-1.6)}{\left[1+\left(\frac{M_w}{10\,000}\right)^{0.6}\right]}$	-2%	1.79	1.80	1.01
	COOH	$\frac{0.12+(0.4-0.12)}{\left[1+\left(\frac{M_w}{40\,000}\right)^{0.6}\right]}$	-4%	0.22	0.23	1.05
	SO_3H	$\frac{1.7+(2.4-1.7)}{\left[1+\left(\frac{M_w}{2\,500}\right)^{0.7}\right]}$	-1%	1.76	1.76	1.00
HWNSSC	OCH_3	$\frac{3.0+(2.2-3.0)}{\left[1+\left(\frac{M_w}{20\,000}\right)^{1.5}\right]}$	+9%	2.51	2.53	1.01
	aliph.OH	$\frac{3.0+(4.9-3.0)}{\left[1+\left(\frac{M_w}{20\,000}\right)^{1.0}\right]}$	-1%	4.12	4.17	1.01
	arom. OH	$\frac{0.9+(1.8-0.9)}{\left[1+\left(\frac{M_w}{25\,000}\right)^{1.0}\right]}$	1%	1.47	1.50	1.02
	COOH	$\frac{0.25+(0.6-0.25)}{\left[1+\left(\frac{M_w}{8\,000}\right)^{2.0}\right]}$	+9%	0.38	0.42	1.11
	SO_3H	$\frac{0.5+(1.4-0.5)}{\left[1+\left(\frac{M_w}{8\,000}\right)^{1.5}\right]}$	+9%	0.85	0.93	1.09

HWKL	OCH_3	$\frac{5.0 + (3.7 - 5.0)}{\left[1 + \left(\frac{M_w}{2\,500}\right)^{2.0}\right]}$	-21%	4.71	4.73	1.00
	aliph.OH	$\frac{0.5 + (1.0 - 0.5)}{\left[1 + \left(\frac{M_w}{16\,000}\right)^{1.4}\right]}$	0%	0.85	0.87	1.02
	arom. OH	$\frac{1.8 + (3.4 - 1.8)}{\left[1 + \left(\frac{M_w}{25\,000}\right)^{1.1}\right]}$	-31%	3.01	3.04	1.01
	COOH	$\frac{0.2 + (0.9 - 0.2)}{\left[1 + \left(\frac{M_w}{10\,000}\right)^{2.0}\right]}$	+267 %	0.62	0.70	1.12
SWKL	OCH_3	$\frac{4.15 + (3.3 - 4.15)}{\left[1 + \left(\frac{M_w}{3\,000}\right)^{2.0}\right]}$	-3%	4.09	4.10	1.00
	aliph.OH	$\frac{2.5 + (1.5 - 2.5)}{\left[1 + \left(\frac{M_w}{10\,000}\right)^{2.5}\right]}$	-6%	2.29	2.32	1.01
	arom. OH	$\frac{2.0 + (4.0 - 2.0)}{\left[1 + \left(\frac{M_w}{150\,000}\right)^{2.0}\right]}$	-8%	3.71	3.77	1.02
	COOH	$\frac{0.27 + (1.5 - 0.27)}{\left[1 + \left(\frac{M_w}{6\,000}\right)^{1.1}\right]}$	-1%	0.52	0.63	1.21
	OH ratio	$\frac{1.0 + (0.5 - 1.0)}{\left[1 + \left(\frac{M_w}{30\,000}\right)^{2.5}\right]}$	+24%	0.75	0.79	1.05

Figure S6. Estimation of functional group contents of ultrafiltrated SWLS (UF10–UF100) and SWKL (F1–F7) fractions based on their MMD. Linear fits are based on the respective SWLS and SWKL fractions from preparative SEC.

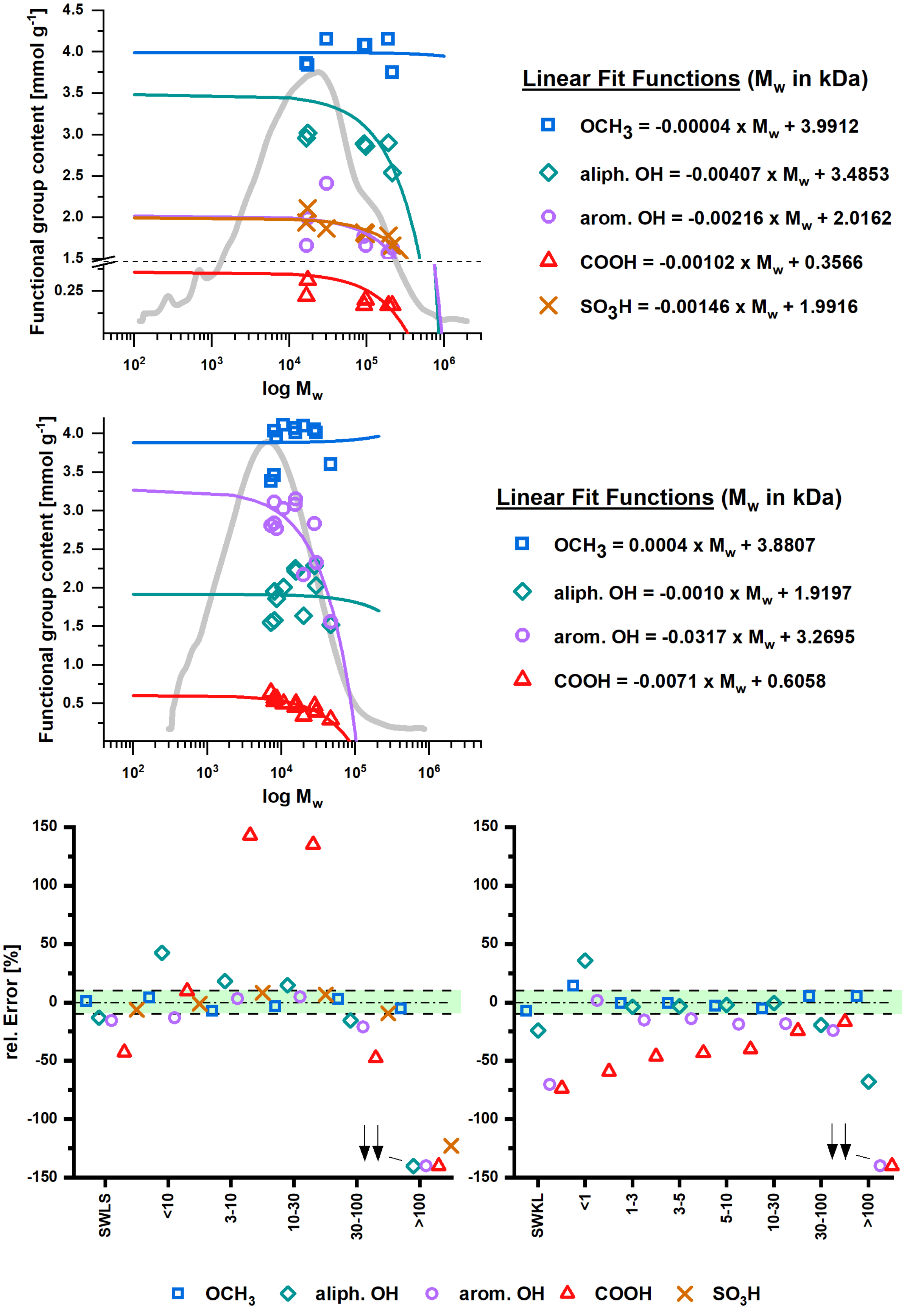

Table S5. Data on SWLS fractions after preparative HIC.[2]

Nr.	Sample	peak area in HIC<br>%	SEC-MALS<br>Mw<br>kDa	EA<br>SO_3H<br>mmol g^{-1}
**1**	1F	11	33.11	1.84
**2**	2F	4	23.12	1.77
**3**	3F	20	40.09	1.58
**4**	4F	50	17.34	1.58
**5**	5F	13	214.60	1.53
**6**	6F	2	277.40	1.38

References

(1) Zinovyev, G.; Sumerskii, I.; Korntner, P.; Sulaeva, I.; Rosenau, T.; Potthast, A. Molar Mass-Dependent Profiles of Functional Groups and Carbohydrates in Kraft Lignin. *J. Wood Chem. Technol.* 2017, *37* (3), 171–183. https://doi.org/10.1080/02773813.2016.1253103.

(2) Musl, O.; Sulaeva, I.; Bacher, M.; Mahler, A. K.; Rosenau, T.; Potthast, A. Hydrophobic Interaction Chromatography in 2 D Liquid Chromatography Characterization of Lignosulfonates. *ChemSusChem* 2020, *13* (17), 4595–4604. https://doi.org/10.1002/cssc.202000849.
